# A Chemical Genetics Strategy That Identifies Small Molecules Which Induce the Triple Response in *Arabidopsis*

**DOI:** 10.3390/molecules22122270

**Published:** 2017-12-19

**Authors:** Keimei Oh, Tomoki Hoshi, Sumiya Tomio, Kenji Ueda, Kojiro Hara

**Affiliations:** 1 Department of Biotechnology, Faculty of Bioresource Sciences, Akita Prefectural University, 241-438, Shimoshinjo Nakano, Akita 010-0195, Japan; timon0822@gmail.com (T.H.); bskt.suns_s.n01056789@docomo.ne.jp (S.T.); 2 Department of Biological Production, Faculty of Bioresource Sciences, Akita Prefectural University, 241-438, Shimoshinjo Nakano, Akita 010-0195, Japan; uken@akita-pu.ac.jp (K.U.); kojiro_h@akita-pu.ac.jp (K.H.)

**Keywords:** ethylene, sulfonamide derivatives, pyrazole derivatives, plant growth regulator

## Abstract

To explore small molecules with ethylene-like biological activity, we conducted a triple response-based assay system for chemical library screening. Among 9600 compounds, we found *N*-[(1,3,5-trimethyl-1*H*-pyrazol-4-yl)methyl]-*N*-methyl-2-naphthalenesulfonamide (**EH-1**) displayed promising biological activity on inducing a triple response in *Arabidopsis* seedlings. Chemical synthesis and structure–activity relationship (SAR) analysis of **EH-1** analogues with different substitution patterns on the phenyl ring structure of the sulfonamide group indicated that 3,4-dichloro-*N*-methyl-*N*-(1,3,5-trimethyl-1*H*-pyrazol-4-yl-methyl)benzenesulfonamide (**8**) exhibits the most potent biological activity. To determine the mechanism of action, we conducted RNA sequencing (RNA-Seq) analysis of the effect of **EH-1** and 1-aminocyclopropane-1-carboxylate (ACC), the precursor of ethylene biosynthesis, following the quantitative real-time polymerase chain reaction (RT-PCR) confirmation. Data obtained from RNA-Seq analysis indicated that **EH-1** and ACC significantly induced the expression of 39 and 48 genes, respectively (above 20 fold of control), among which five genes are up-regulated by **EH-1** as well as by ACC. We also found 67 and 32 genes that are significantly down-regulated, respectively, among which seven genes are in common. For quantitative RT-PCR analysis. 12 up-regulated genes were selected from the data obtained from RNA-Seq analysis. We found a good correlation of quantitative RT-PCR analysis and RNA-Seq analysis. Based on these results, we conclude that the action mechanism of **EH-1** on inducing triple response in *Arabidopsis* is different from that of ACC.

## 1. Introduction

The process of seeds growing into plants is one of the most dynamic biological processes in a plant’s life cycle. The life of the buried seedlings often starts in the subterranean darkness when water is provided as long as the temperature is appropriate. Etiolation is the process that enables seedlings to emerge from soil. Subsequent skotomorphogenesis makes seedlings acquire autotrophic ability through the formation of the photosynthetic apparatus. These biological events, however, require seedlings to be able to sense any mechanical obstacles in soil so as to protect them from wounding. Hence, inhibition of the rapid elongation of the etiolated hypocotyl is needed to optimize the seedlings to push through the soil without damaging their shoot meristems. Over time and through adaptation, plant seedlings have evolved an elaborate mechanism during these processes. Seedlings produce the phytohormone ethylene thereby increasing their soil penetration ability. In the presence of ethylene, seedlings grown in the darkness display a thickened hypocotyl and an exaggerated apical hook. This effect of ethylene in dark-grown seedlings is called ‘triple response’ [1]. Available evidences indicated that ethylene plays a crucial role in the process of seedlings’ dark growth.

Ethylene is a naturally occurring gaseous plant hormone that plays key roles in regulating broad aspects of physiological processes in plants [2,3]. Ethylene has been implicated in developmental processes such as the formation of the apical hook in dark-grown seedlings, the regulation of flower development, fruit growth and ripening [4,5,6,7]. Ethylene also regulates plant responses to biotic and abiotic stresses including pathogen inflection, flooding and/or drought [8,9,10].

Because ethylene affects several important agronomy traits such as senescence and fruits ripening, great efforts have been made to develop technologies for the manipulation of ethylene levels in plant tissues by using chemicals. Ethephon (IUPAC name: 2-chloroethylphosphonic acid), a prodrug of ethylene which degrades and subsequently releases ethylene in plant tissues, is a commercially available and the most widely used plant growth regulator [11]. Ethephon has been used for promoting fruit ripening, abscission and flower induction [12]. Ethephon has also been registered as pesticide on a number of food, feed and nonfood crops such as cotton.

By now, great efforts have been made to develop chemicals with ethylene activity. To meet the demands of developing new chemicals which are non-gaseous at normal atmosphere but with ethylene-like activity, we conducted a chemical genetics approach to search for such compounds. Currently, chemical genetics is an effective way to identify chemicals with biological activity [13,14,15]. This method is based on the phenotypic screening of chemical compound libraries to find the chemicals that are able to induce phenotypic changes in plants in a bioassay system.

A bioassay, which is known as the ‘triple response’ assay, has been demonstrated quite useful to determine the effect of ethylene in plants [1,16]. By now, many key components of the ethylene signal transduction pathway have been identified by using a ‘triple response’ assay in genetic screening. ETR1 and ETR2, the ethylene receptors, were identified by using a triple response assay [17,18]. CTR1 which is a negative regulator of the ethylene signaling pathway [19], and other components were also identified by using triple response assays [20,21,22]. In this context, the ‘triple response’ assay is useful in identification chemicals with ethylene activity in compound library screening.

In the present work, we report the screening of a chemical compound library and the discovery of a new small molecule that displays promising activity in inducing triple response morphological characteristics in *Arabidopsis* seedlings. The discovered compound was named **EH-1**. Chemical synthesis of analogues of **EH-1** was conducted in the present work. We also conducted structure-activity relationship studies to determine the chemical structure impact of this synthetic series on induction of the triple response in *Arabidopsis* seedlings. Using RNA sequencing (RNA-seq) techniques combined with quantitative real-time polymerase chain reaction (RT-PCR) analysis, we also determined the mode of action of **EH-1**.

## 2. Results

### 2.1. Screening of a Chemical Library for Small Molecules with Triple Response Induction Activity

**Figure 1:**
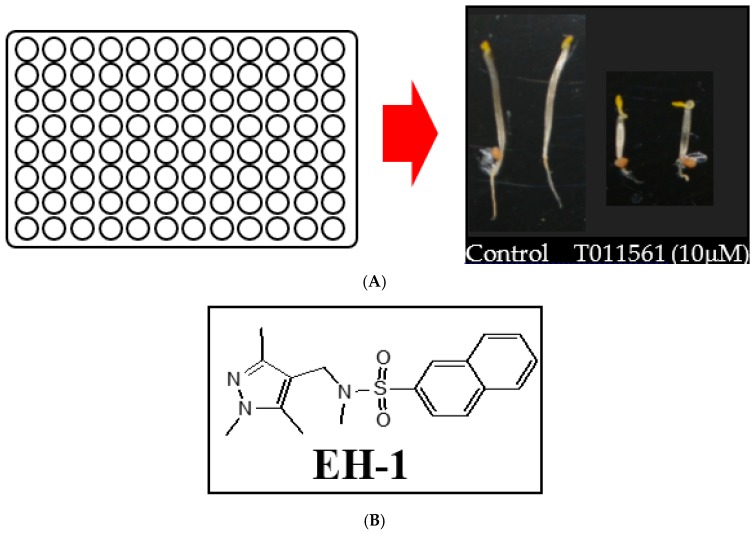
A screen for chemicals with activity to induce triple response in *Arabidopsis* seedlings. (**A**) Approximately 10 seeds/well of *Arabidopsis* were grown for five days in the dark in 96 well plates on medium containing ½ MS. We use dimethyl sulfoxide (DMSO) mock treatment as a control and 1-aminocyclopropane-1-carboxylate (ACC) (10 μM) as a positive control. Seedlings with short hypocotyls and exaggerated apical were marked as positive hits while the root length were not determined in the library screening. Among 9600 compounds (10 μM). One compound (**EH-1**) displayed biological activity; (**B**) The chemical structure of **EH-1**.

To identify small molecules displaying triple response induction activity, we screened a diverse set of 9600 synthesized chemicals obtained from Drug Discovery Initiative at the University of Tokyo [23] using a triple response assay in *Arabidopsis* seedlings grown in the dark. The seeds were placed in 96-well plates containing half-strength Murashige and Skoog (MS) agar medium and individual chemicals was adjusted at a final concentration of 10 μM in each well. Plants were grown in the dark for five days at room temperature and the biological activity of the compounds were examined visually by a method we previously described [24]. The biological activity of test compounds on inducing triple response was established by determining the activity of chemicals on inhibition the hypocotyl length, root, and measuring the angle of the apical hook of *Arabidopsis* seedlings (Figure 1). *Arabidopsis* seedlings exhibiting short hypocotyls and with an exaggerated apical hook were marked as positive hits. We found 35 compounds that caused short hypocotyls, among which six compounds also displayed different degrees of exaggerated apical hook.

### 2.2. Identification of ***EH-1***

After retesting, one compound, a sulfonamide derivative, displayed biological activity on inducing short hypocotyls and exaggerated apical hook of *Arabidopsis* seedlings grown in the dark (Compound T011561, Figure 1A). As shown in Figure 1A, plants grown in the presence of 10 μM of compound T011561 displayed short hypocotyls as well as an exaggerated apical hook in comparison to those plants grown in the DMSO mock treatment (Figure 1A, picture). The hypocotyls of T011561-treated plants (Figure 1A, two plants on the right) were approximately one third of the height of those of the DMSO-treated control (Figure 1A, two plants on the left). The degree of apical hook for those plants grown in DMSO were found to be approximately 58 ± 16 degrees. In contrast, the degree of apical hook of the plants grown in the presence of T0111561 (10 μM) was found to be approximately 205 ± 18 degrees. In terms of root length, the DMSO-treated control plants were approximately 1.7 ± 0.2 mm long, while the root length of plants grown in T011561 was found to be approximately 0.5 ± 0.1 mm. This result clearly indicated that compound T0111561 induces the morphological characteristics of triple response which is similar to that of ethylene: with short hypocotyls and root, and an exaggerated apical hook. The IUPAC name for compound T011561 is *N*-[(1,3,5-trimethyl-1*H*-pyrazol-4-yl)methyl]-*N*-methyl-2-naphthalenesulfonamide. We named the compound **EH-1**.

### 2.3. Chemical Synthesis of ***EH-1*** Analogues

**Scheme 1:**
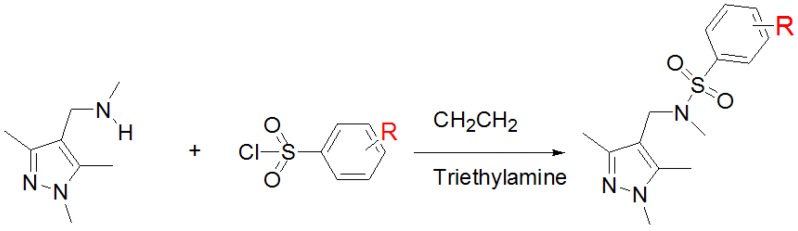
General synthetic route for preparing the analogues of **EH-1**.

To further confirm the biological activity of **EH-1** on induction of triple response in *Arabidopsis*, we prepared **EH-1** and synthesized some of its analogues. The analogues of **EH-1** were designed based on the replacing the naphthalene moiety in **EH-1** with a phenyl moiety containing different substituents and substitution patterns (Scheme 1). The synthesis of **EH-1** analogues was achieved by using commercially available *N*-methyl-1-(1,3,5-trimethyl-1*H*-pyrazole-4-yl)methanamine as a starting material. Scheme 1 outlines the general synthetic route for preparation of the sulfonamide target compounds. Table 1 lists the chemical structures of the compounds prepared in the present work.

### 2.4. Triple Response Effect of ***EH-1*** Analogues in Arabidopsis Seedlings

**Figure 2:**
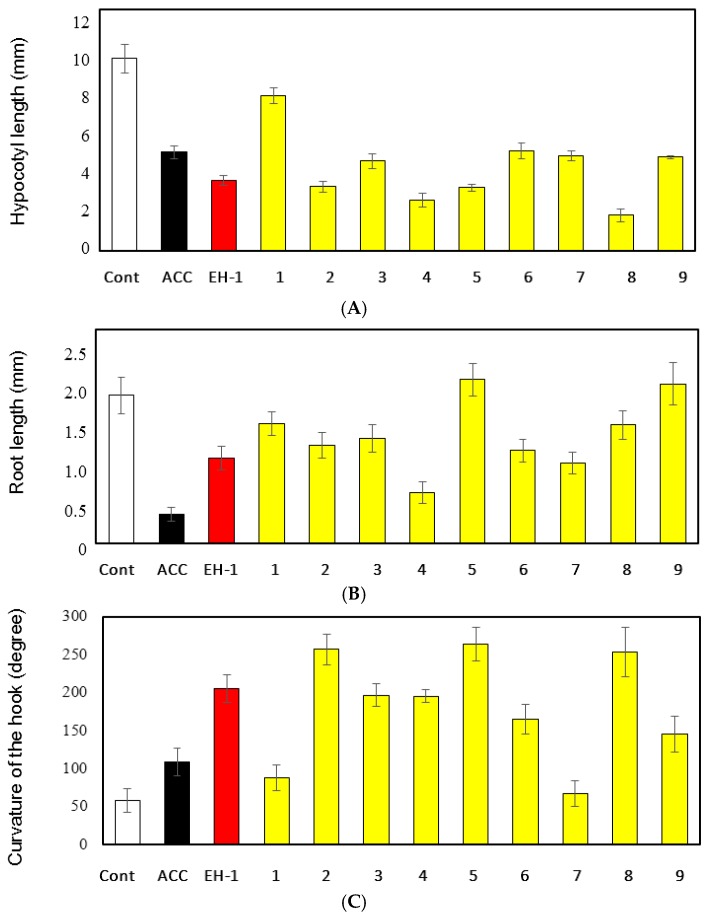
Effect of EH-1 and its analogues on inducing morphology typical of triple response in *Arabidopsis* seedlings. The triple responses of *Arabidopsis* seedlings were measured by determining the hypocotyl length, root length and curvature of the hook. Approximately 50 seeds/well of *Arabidopsis* were grown for five days in the dark in a 24 well plate on medium containing ½ MS and the concentration of the test compounds including ACC and **EH-1** are set at a final concentration of 10 μM. ACC was used as a positive control while using DMSO mock treatment as a control. The chemical structures of the test compounds (in number) were as shown in Table 1. (**A**) Effect of sulfonamide derivatives on hypocotyl elongation; (**B**) Effect of sulfonamide derivatives on root growth; (**C**) Effect of sulfonamide derivatives on inducing exaggerated apical hook of *Arabidopsis* seedlings were measured by determine 11 seeds. Data are the means ± s.e. obtained from 11 to 15 plants. All the experiments were done three times to establish the repeatability.

Next, we determined the effect of the compounds synthesized in the present work on induction of triple response in *Arabidopsis* seedlings while using 1-aminocyclopropane-1-carboxylate (ACC) as a positive control. We first determined the effect of the **EH-1** and its analogues on stem elongation of *Arabidopsis* seedlings grown in the dark. As shown in Figure 2A, the hypocotyl length of the non-chemical treated control was approximately 9.5 ± 0.72 mm (the white bar) while the hypocotyl length of ACC (10 μM)-treated *Arabidopsis* seedlings was approximately 4.9 ± 0.32 mm (the black filled bar). This result indicated that ethylene inhibits the stem elongation of *Arabidopsis* seedlings in our assay system. In terms of the biological activity of the synthesized **EH-1**, we found that **EH-1** (10 μM) reduced the hypocotyl length of *Arabidopsis* seedlings from 9.5 ± 0.72 mm (the white bar) to 3.5 ± 0.24 mm (the red bar). This result indicated that the inhibitory potency of **EH-1** on stem elongation of *Arabidopsis* seedlings is stronger than ACC. Introducing a phenyl ring instead of a naphthalene moiety (compound **1**) weakened the inhibitory potency with hypocotyl length of *Arabidopsis* seedlings to approximately 7.7 ± 0.39 mm (the first yellow bar from the left). This result indicated that reducing the size of aromatic ring structure at this position has a negative effect on promoting the inhibitory activity of stem elongation of *Arabidopsis* seedlings. Introducing a chlorine atom at position 4 or position 3 of the phenyl ring (compounds **2**, **3**) enhanced the inhibitory activity in comparison with compound **1** with hypocotyl length approximately 3.2 ± 0.27 mm and 4.5 ± 0.36 mm, respectively. Introduction of two chlorine atoms (compounds **4**–**9**) at different positions of the phenyl ring displayed different effects on inhibition of stem elongation of *Arabidopsis* seedlings. Among the compounds prepared in the present work, we found that compound **8** with two chlorine atoms at positions 3 and 4 of phenyl ring displayed the most potent inhibitory activity with a hypocotyl length of approximately 1.8 ± 0.32 mm (the second yellow bar from the right). Next, we determined the effect of **EH-1** and its analogues on inhibition root elongation. As shown in Figure 2B, the root length of the non-chemical treated control was approximately 1.7 ± 0.2 mm (the white bar) while the hypocotyl length of ACC (10 μM) treated *Arabidopsis* seedlings were found approximately 0.3 ± 0.1 mm (the black filled bar). This result indicated that ethylene inhibits the root elongation of *Arabidopsis* seedlings in our assay system. The biological activity of the chemicals prepared in the present work, we found that **EH-1** (10 μM) reduced the root length of *Arabidopsis* seedlings from 1.7 ± 0.2 mm (the white bar) to 1.2 ± 0.2 mm (the red bar). This result indicated that the inhibitory potency of **EH-1** on root elongation of *Arabidopsis* seedlings is weaker than that of ACC.

However, when a phenyl ring was introduced instead of a naphthalene moiety (compound **1**), we found that the root length of the *Arabidopsis* seedlings was approximately 1.8 ± 0.39 mm. This result indicated that the analogue with a phenyl ring at the sulfonylamide moiety does not inhibit root elongation significantly. Introducing a chlorine atom at position 4 or position 3 of the phenyl ring (compounds **2**, **3**) enhanced the inhibitory activity on root elongation in comparison with compound **1** with root lengths of approximately 1.4 ± 0.2 mm and 1.1 ± 0.1 mm, respectively. Introduction of two chlorine atoms (compounds **4**–**9**) at different positions of the phenyl ring displayed different effects on root elongation of *Arabidopsis* seedlings. Compound **4** displays a potent inhibitory activity on root elongation (Figure 2B) while compounds **5**, **8** and **9** do not exhibit significant inhibitory activity on root elongation.

Finally, we determined the effect of the **EH-1** and its analogues on apical hook development. As shown in Figure 2C, the curvature of the hook of the non-chemical treated control was approximately 58 ± 16 degrees (the white bar) while the curvature of the hook of ACC (10 μM)-treated *Arabidopsis* seedlings were approximately 109 ± 19 degrees (the black filled bar). This result indicated that ACC induced an exaggerated apical hook in *Arabidopsis* seedlings. In terms of the biological activity of the **EH-1** and its analogues synthesized in the present work, we found that the curvature of the hook of the *Arabidopsis* seedlings treated with **EH-1** (10 μM) were 205 ± 19 degrees (the red bar). This result indicated that **EH-1** exhibits potent activity in inducing an exaggerated apical hook in *Arabidopsis* seedlings. After introducing a phenyl ring instead of the naphthalene moiety (compound **1**), however, we found that the curvature of the hook of the *Arabidopsis* seedlings was approximately 88 ± 17 degrees (the first yellow bar from the left). This result indicated that downsizing the naphthalene moiety had a negative effect on enhancing the biological activity. Introducing a chlorine atom at position 4 or position 3 of the phenyl ring (compounds **2**, **3**) significantly enhanced the biological activity in inducing an exaggerated apical hook of *Arabidopsis* seedlings. The degrees of apical hook were approximately 257 ± 20 degrees and 197 ± 15 degrees, respectively. Introduction of two chlorine atoms (compounds **4**–**9**) at different positions of the phenyl ring produced different effects on inducing an exaggerated apical hook of *Arabidopsis* seedlings. Except for compound **7** which is an analogue with a 2,6-dichlorophenyl moiety that did not display significant biological activity, the other compounds exhibited promising biological activity for inducing an exaggerated apical hook in *Arabidopsis* seedlings. Among all the **EH-1** analogues, we found compounds **2**, **5** and **8** displayed potent activity (the general images of *Arabidopsis* seedlings grown in the presence of 10 μM chemicals or DMSO mock-treated control shown in Figure 2 are given in Figures S1–S12).

### 2.5. RNA-Seq Analysis of the Effect of ***EH-1*** on Arabidopsis Seedlings

**Figure 3:**
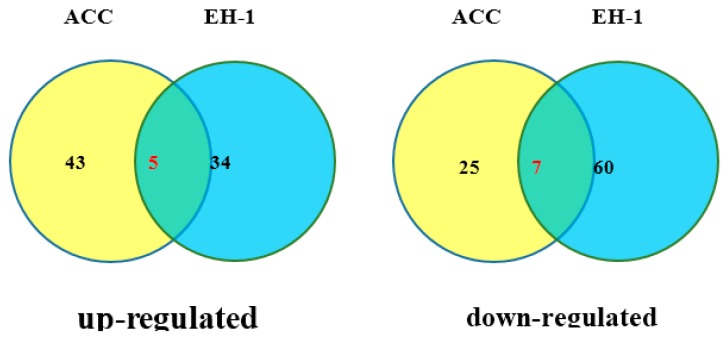
Venn diagram of genes regulated by ACC and **EH-1**. The expression level of genes in *Arabidopsis* seedlings were identified by RNA sequencing (RNA-Seq) analysis. Both up-regulated and down-regulated genes with above 20 fold were estimated from *Arabidopsis* seedlings grown in the plate containing ACC or **EH-1** using no-chemical treated *Arabidopsis* seedlings as a control.

To determine the mode of action of **EH-1**, we conducted three independent trails of transcript expression analysis of RNA-Seq experiments [25]. *Arabidopsis* seedlings used for experiments were grown in half-strength MS agar medium containing **EH-1** (10 μM), ACC (10 μM) and without chemical treatment, respectively. Data obtained from the RNA-Seq experiments indicated that a total of 156.9 million paired-end reads with a read length of 101 bp were generated by next-generation sequencer (HiSeq 1000, Illumina Inc., San Diego, CA, USA). The preprocessed high-quality 148.3 million reads pairs were aligned to a TAIR10 gene model thereby determining the differentially expressed genes (DEGs). We found that the number of over 20-fold up-regulated DEGs in *Arabidopsis* seedlings with **EH-1** and ACC treatment were 39 and 48, respectively (Figure 3 left, Table S1). Simultaneously, we found that the number of over 20-fold down-regulated DEGs in *Arabidopsis* seedlings with **EH-1** or ACC treatment were 67 and 32, respectively (Figure 3 right, Table S1). Several well-known ethylene responsive marker genes such as extensin and peroxidase [26,27] were detected in our *Arabidopsis* seedlings grown in plates containing ACC (10 μM). However, these genes were not detected in *Arabidopsis* seedlings grown in the plate containing **EH-1** (10 μM). This result suggests that the mode of action of **EH-1** may be different from ACC on inducing triple response of *Arabidopsis* seedlings as a consequence of the fact the pattern of the gene expression profiles induced between ACC and **EH-1** are different. Another line of evidence obtained from our RNA-Seq experiments was that ACC and **EH-1** commonly induce and/or reduce the expression level of several genes (Figure 3). These genes are possible important clues of identify the mode of action of **EH-1** on inducing triple response in *Arabidopsis* seedlings.

### 2.6. Quantitative RT-PCR Analysis of the Genes Induced by ***EH-1*** and ACC

To validate the reliability of the expression profiles obtained from RNA-Seq analysis, we conducted real-time quantitative RT-PCR analyses. In the present work, we selected 12 genes which are significantly up-regulated by **EH-1** and/or ACC in RNA-Seq analysis for real-time quantitative RT-PCR analysis. Six genes which are significantly up-regulated by **EH-1** were selected for validation the **EH-1** expression profiles obtained from RNA-Seq analysis. As shown in Table 2, genes display high up-regulated rates from 36.33 to 134.32 estimated by RNA-Seq analysis (Table 2, third column). Data obtained from qRT-PCR analysis (Table 2, fourth column) displayed a high rate of the expression level of these genes from 14.65 ± 7.47 to 97.13 ± 49.08. This result is in good correlation with the data obtained from RNA-Seq analysis, indicating the gene expression profiles of **EH-1** obtained from RNA-Seq analysis have been validated by real-time quantitative RT-PCR analysis.

Next, we selected another six genes which were highly up-regulated by ACC treatment for real-time quantitative RT-PCR analysis. Among these genes, one gene (AT1G72290) is also found to be significantly up-regulated by **EH-1** with a rate of approximately 134.32 (see Table 2) in our RNA-Seq analysis. Table 2 displayed the data obtained from RNA-Seq analysis (Table 2, third column) and real-time quantitative RT-PCR analysis. (Table 2, fourth column). Our data obtained from real-time quantitative RT-PCR analysis are in good correlation with the data obtained from RNA-Seq analysis, indicating the gene expression profile of ACC obtained from RNA-Seq analysis have been validated by real-time quantitative RT-PCR analysis.

## 3. Discussion

In the present work, we carried out a chemical genetic screening of small molecules displaying biological activity on inducing triple response effects in *Arabidopsis* seedlings. We found a sulfonamide derivative **EH-1** that exhibits promising activity. We confirmed the triple response-inducing biological activity of **EH-1**, not only through chemical synthesis of this chemical by a method we designed, but also by using its analogues. Taking these results together, our data clearly indicated that most of the analogues prepared in this work display promising activity in inducing the triple response effect in *Arabidopsis* seedlings. Replacing the naphthalene moiety in **EH-1** with a phenyl ring had a negative effect on enhancing the biological activity. However, introducing chlorine atom(s) on the phenyl ring gave a significant effect in reducing or enhancing the biological activity. For example, compound **4** displays promising inhibitory activity on hypocotyl length as well as root length, but the potency for inducing exaggeration of the apical hook is weaker than that of compounds **2**, **5** and **8**. Compound **5** inhibits hypocotyl length effectively and also shows more exaggeration of the apical hook, however, the length of the roots were not different from mock-treated roots. Compound **7** on the other hand is effective in inhibiting hypocotyl elongation and root elongation, but the apical hooks seem no different from control. Based on the data obtained from the present work, we found that compound **8** which is a 3,4-dichlorobenzenesulfonamide analogue displayed the most potent activity on inducing the triple response in *Arabidopsis* seedlings. Our results suggest that chemical modification the sulfonamide moiety greatly influence the biological activity of this synthetic series on induction of triple response in *Arabidopsis*.

We thus have now discovered a new series of sulfonamide derivatives that exhibit promising biological activity in inducing triple response in *Arabidopsis* seedlings. Data obtained indicated that the substitution of the phenyl moiety in this synthetic series significantly affects the biological activity. Further studies on the chemical modification and structure-activity relationship studies including the pyrazole moiety of this synthetic series, which is currently undergoing in our laboratory, may lead to the discovery of more potent compounds as novel plant growth regulators.

To determine the mode of action of this synthetic series, we carried out RNA-seq analysis combined with real-time quantitative RT-PCR analysis to determine the expression levels of the genes affected by **EH-1** and ACC. Data obtained from quantitative RT-PCR analysis indicated a good correlation with RNA-seq analysis (Table 2). Thus, it is possible allow us to determine the similarity of the gene profiles induced by **EH-1** and ACC by using our RNA-seq analysis data. Our results indicated that **EH-1** up-regulated expression of 39 genes by above 20 fold compared to that of non-chemically treated control while ACC up-regulated 48 genes in *Arabidopsis* seedlings. Among these genes, only five genes were found commonly up-regulated by ACC and **EH-1** (Figure 3). This result indicated that only approximately 13% of the genes were up-regulated by **EH-1** while approximately 10% of those genes for ACC are in common. Similarly, among genes which are down-regulated by **EH-1** and/or ACC, the percentage of the genes commonly down-regulated was less than 22% (Figure 3). Taking these results together, our results clearly indicate that the gene profiles induced by **EH-1** and ACC in *Arabidopsis* seedlings are quite different. Thus, our results negate the possibility that **EH-1** induces a triple response effect in *Arabidopsis* seedlings due to the action of **EH-1** on induction of ethylene stimulation in plant tissue. Our results also suggest that the mechanism of action **of EH-1** to induce triple response in *Arabidopsis* seedlings may be different from that of ethylene. Using RNA-seq techniques, we detected the expression levels of the genes which are significantly induced or reduced by the treatment of **EH-1** and ACC. Data obtained from our experiment indicated that gene profiles induced or reduced by **EH-1** are quite different from those of ACC. Information about whether **EH-1** stimulates the production of ethylene still needs to be further determined. Also, establishment of an efficient gas chromatography (GC) method to quantify the ethylene in *Arabidopsis* seedlings is important to explore the mechanism of action of **EH-1**. To identify the mechanism of action **of EH-1**, the gene profile induced by **EH-1** which has been determined in the present work is an important clue. Also, **EH-1** is a possible experiment tool for dissecting the molecular mechanism of triple response as well as the action of ethylene. Using ethylene signaling mutants may be a useful approach to determine the mechanism of action of **EH-1**. We expect further studies on the application of **EH-1** may lead to the discovery of novel plant growth regulators.

## 4. Materials and Methods 

### 4.1. General

Chemicals for synthesis were purchased from Kanto Chemicals Co. Ltd. (Tokyo, Japan) and Tokyo Kasei Co. Ltd. (Tokyo, Japan). Reagents were of the highest grade commercially available. ^1^H-NMR spectra were recorded with a ECP-400 spectrometer (JEOL, Tokyo, Japan), chemical shifts being expressed in ppm downfield from TMS as an internal standard. High resolution Electrospray Ionization Fourier Transform Ion Cyclotron Resonance mass spectra (ESI-FTICR) were recorded on an Exactive MS System (Thermo Fisher Scientific, Waltham, MA, USA).

### 4.2. Chemical Compound Library and Chemicals for Biological Studies

The chemical compound library was obtained from the Drug Discovery Initiative (The University of Tokyo, Tokyo, Japan). A diverse subset of 9600 compounds was assigned as a core chemical library with varieties of structural diversity. The structure of the compound was identified by liquid chromatography-mass spectrometry (LC-MS), and the purity was checked by the signal of the evaporative light-scattering detector (ELSD) [23]. Stock solutions of the test compounds were dissolved in DMSO at a concentration of 100 mM and stored at −30 °C before use.

### 4.3. Chemical Synthesis: General Procedure of Preparation of N-Methyl-N-[(1,3,5-trimethyl-1H-pyrazol-4-yl)methyl]-2-naphthalenesulfonamide *(**EH-1**)*

2-Naphthalenesulfonyl chloride (0.26 mmol) was added to a 0.2 M methylene chloride solution of trimethylamine (1.0 mmol) and *N*-methyl-1-(1,3,5-trimethyl-1*H*-pyrazole-4-yl)methanamine (0.26 mmol), and stirred at room temperature for 12 h. The reaction mixture was washed with 5% NaHCO_3_, 1 N HCl and saturated NaCl. The organic layer was dried and the solvent was removed under the reduced pressure. The residue was purified by chromatography using CHCl_3_:MeOH = 9:1 as an elution solution to obtain 68.5 mg of the target compound (Yield: 76.8%). ^1^H-NMR (CDCl_3_) δ 2.02 (s, 3H), 2.05 (s, 3H), 2.62 (s, 3H), 3.65 (s, 3H), 4.07 (s, 2H), 7.55–7.69 (m, 3H), 7.94 (d, *J* = 8.5 Hz, 1H), 8.10 (d, *J* = 5.4 Hz, 1H), 8.22 (d, *J* = 4.0 Hz, 1H), 8.83 (d, *J* = 5.4 Hz, 1H). HRMS: Calculated for: C_30_H_34_N_3_O_2_S [M + H]^+^ = 344.1433, Found: 344.1438. Other compounds **1**–**9** were prepared in a similar way by the reaction of *N*-methyl-1-(1,3,5-trimethyl-1*H*-pyrazole-4-yl)methanamine with the corresponding benzenesulfonyl chloride.

*N-Methyl-N-[(1,3,5-trimethyl-1H-pyrazol-4-yl)methyl]benzenesulfonamide* (**1**): ^1^H-NMR (CDCl_3_) δ 2.16 (s, 3H), 2.20 (s, 3H), 2.49 (s, 3H), 3.69 (s, 3H), 3.89 (s, 2H), 7.54–7.62 (m, 3H), 7.81–7.83 (m, 2H). HRMS: Calculated for: C_14_H_20_N_3_O_2_S [M + H]^+^ = 294.1276, Found: 294.1262.

*4-Chloro-N-methyl-N-[(1,3,5-trimethyl-1H-pyrazol-4-yl)methyl benzenesulfonamide* (**2**): ^1^H-NMR (CDCl_3_) δ 2.14 (s, 3H), 2.21 (s, 3H), 2.50 (s, 3H), 3.71 (s, 3H), 3.90 (s, 2H), 7.55 (d, *J* = 5.5 Hz, 2H), 7.77 (d, *J* = 5.5 Hz, 2H). HRMS: Calculated for C_14_H_19_ClN_3_O_2_S [M + H]^+^ = 328.0886. Found: 328.0891.

*3-Chloro-N-methyl-N-[(1,3,5-trimethyl-1H-pyrazol-4-yl)methyl benzenesulfonamide* (**3**): ^1^H-NMR (CDCl_3_) δ 2.16 (3H, s), 2.23 (3H, s), 2.53 (3H, s), 3.73 (3H, s), 3.92 (2H, s), 7.52 (1H, t, *J* = 7.9), 7.61 (1H, d, *J* = 8.0), 7.71 (1H, d, *J* = 7.8), 7.81 (1H, s). HRMS: C_14_H_19_ClN_3_O_2_S [M + H]^+^ = 328.0886. Found: 328.0878.

*2,3-Dichloro-N-methyl-N-(1,3,5-trimethyl-1H-pyrazol-4-ylmethyl)-benzenesulfonamide* (**4**): ^1^H-NMR (CDCl_3_) δ 2.15 (3H, s), 2.20 (3H, s), 2.68 (3H, s), 3.70 (3H, s), 4.22 (2H, s), 7.35 (1H, t, *J* = 8.0), 7.68 (1H, dd, *J*_1_ = 1.4, *J*_2_ = 8.0), 8.00 (1H, dd, *J*_1_ = 1.6, *J*_2_ = 8.0). HRMS: Calculated for C_14_H_18_Cl_2_N_3_O_2_S [M + H]^+^ = 362.0497. Found: 362.0492.

*2,4-Dichloro-N-methyl-N-(1,3,5-trimethyl-1H-pyrazol-4-ylmethyl)benzenesulfonamide* (**5**): ^1^H-NMR (CDCl_3_) δ2.16 (3H, s), 2.21 (3H, s), 2.66 (3H, s), 3.70 (3H, s), 4.18 (2H, s), 7.38 (1H, dd, *J*_1_ = 2.1, *J*_2_ = 8.4), 7.56 (1H, d, *J* = 2.1), 7.98 (1H, d, *J* = 8.4). HRMS: Calculated for C_14_H_18_Cl_2_N_3_O_2_S [M + H]^+^ = 362.0497. Found: 362.0502.

*2,5-Dichloro-N-methyl-N-(1,3,5-trimethyl-1H-pyrazol-4-ylmethyl)-benzenesulfonamide* (**6**): ^1^H-NMR (CDCl_3_) δ 2.16 (3H, s), 2.23 (3H, s), 2.71 (3H, s), 3.73 (3H, s), 4.21 (2H, s), 7.46–7.49 (1H, m). HRMS: Calculated for C_14_H_18_Cl_2_N_3_O_2_S [M + H]^+^ = 362.0497. Found: 362.0486.

*2,6-Dichloro-N-methyl-N-(1,3,5-trimethyl-1H-pyrazol-4-ylmethyl)benzenesulfonamide* (**7**): ^1^H-NMR (CDCl_3_) δ 2.01 (s, 3H), 2.06 (s, 3H), 2.63 (s, 3H), 3.66 (s, 3H), 4.07 (s, 2H), 7.55–7.68 (m, 3H), 7.95 (d, *J* = 8.5 Hz, 1H), 8.09 (d, *J* = 5.3 Hz, 1H), 8.22 (d, *J* = 4.0 Hz, 1H), 8.81 (d, *J* = 5.3 Hz, 1H). HRMS: Calculated for C_14_H_18_Cl_2_N_3_O_2_S [M + H]^+^ = 362.0497. Found: 362.0481.

*3,4-Dichloro-N-methyl-N-(1,3,5-trimethyl-1H-pyrazol-4-ylmethyl)benzenesulfonamide* (**8**): ^1^H-NMR (CDCl_3_) δ 2.14 (s, 3H), 2.21 (s, 3H), 2.50 (s, 3H), 3.71 (s, 3H), 3.90 (s, 2H), 7.55 (d, *J* = 5.5 Hz, 2H), 7.77 (d, *J* = 5.5 Hz, 2H). HRMS: Calculated for C_14_H_18_Cl_2_N_3_O_2_S [M + H]^+^ = 362.0497. Found: 362.0478.

*3,5-Dichloro-N-methyl-N-(1,3,5-trimethyl-1H-pyrazol-4-ylmethyl)benzenesulfonamide* (**9**): ^1^H-NMR (CDCl_3_) δ 2.14 (s, 3H), 2.21 (s, 3H), 2.50 (s, 3H), 3.71 (s, 3H), 3.90 (s, 2H), 7.55 (d, *J* = 5.5 Hz, 2H), 7.77 (d, *J* = 5.5 Hz, 2H). HRMS: Calculated for C_14_H_18_Cl_2_N_3_O_2_S [M + H]^+^ = 362.0497. Found: 362.0508.

### 4.4. Plant Materials and Growth Conditions

Seeds of *Arabidopsis* (ecotype Columbia) were purchased from Lehle Seeds (Round Rock, TX, USA). Seeds used for the assay were sterilized in 1% NaOCl for 20 min and washed with sterile distilled water. Seeds (approximately 50) were sown on a 1% solidified agar medium containing 1/2 Murashige and Skoog (MS) salt added to 96-well plates (Fukaekasei Co., Ltd., Kobe, Japan) for library screening and to 24 well plates (Fukaekasei Co., Ltd.) for test the biological activity of synthesized compounds. The plates were wrapped with three layers of aluminum foil to keep the seed germination in a dark condition. After pre-incubation the plates at 4 °C for two days, plants were grown in a growth chamber at 25 °C for 5 days. Effect of the test compounds on growth of *Arabidopsis* seedlings were determined by measuring the hypocotyl length, root length and degree of the apical hook. Stock solutions of all of the chemicals were dissolved in DMSO and stock at −30 °C before use. The amount of DMSO was added below 0.1% (*v*/*v*) of growth media in all the experiments.

### 4.5. Isolation of Total-RNA

The seedlings were collected from the 90-mm diameter plastic petri dishes under the above growth conditions. Total RNA was extracted from the tissues using the RNeasy Plant Mini Kit (Qiagen, Tokyo, Japan). To avoid amplifying genomic DNA, each RNA fraction was treated with RNase-free DNaseI (RQ1; Promega KK, Tokyo, Japan). DNA elimination was confirmed by a usual RT-PCR with or without the reverse transcriptase. The isolated total RNA samples were stored −70 °C before use.

### 4.6. RNA-Seq Analysis

Triplicate cDNA libraries for each treatment were prepared and sequenced with 100 bp paired-end reads by the Illumina HiSeq 1000 platform at the Biotechnology Center in Akita Prefectural University. Raw data were preprocessed by three programs (FASTX-Toolkit, Prinseq and Trimmomatic). RNA-seq analysis was carried out using the Tophat-Cufflinks pipeline [28], with the following versions: Tophat v2.0.14, Bowtie2 v2.2.6.0 and Cufflinks v2.2.1. The *Arabidopsis* TAIR10 genome and gene model annotation data were downloaded and used for reference. Differentially expressed genes (DEGs) were determined by applying the screening thresholds of 20-fold changes in FPKM (fragments per kilobase of exon per million fragments mapped) and FDR-adjusted *p*-value ≤ 0.05 using Cuffdiff tool.

### 4.7. qRT-PCR Analysis

The real-time qRT-PCR was performed in a CFX96 C1000 Thermal Cycler (Bio-Rad Laboratories, Tokyo, Japan), using an iTaq Universal SYBR Green One-Step Kit (Bio-Rad). The reaction mixture (10 μL) contained 0.3 μM each forward and reverse primer, 1× iTaq universal SYBR Green reaction mix, 0.125 μL iScript reverse transcriptase, and 10 ng total RNA. The gene specific primer sets are listed in Table S2, and actin gene was used as an endogenous control. The RT-PCR amplification profile was as follows: cDNA synthesis for 10 min at 50 °C, followed by reverse transcriptase inactivation for 1 min at 95 °C, then 40 cycles of denaturation at 95 °C for 10 s and anneal/extension at 60 °C for 30 s.

### 4.8. Statistical Analysis

All measurements were carried out at least in triplicate. Data analysis (*t*-test and analysis of variance) was applied to determine the significant difference with the use of significance throughout the manuscript being based upon *p* < 0.05 unless stated otherwise.

## 5. Conclusions 

We have discovered a sulfonamide derivative **EH-1** that indices the triple response in *Arabidopsis* seedlings through compound library screening. To confirm the biological activity of **EH-1**, we designed a chemical synthetic route and synthesized **EH-1** together with nine analogues. Data obtained from biological studies indicated that **EH-1** and its analogues exhibited promising activity in inducing triple response in *Arabidopsis* seedlings. Data obtained from RNA-seq analysis combined with the confirmation of qRT-PCR analysis indicated that the mode of action on triple response induction of **EH-1** may be different from ethylene.

## Figures and Tables

**Table 1:** The chemical structures of **EH-1** and its analogues.

Compound No.	Chemical Structure	Compound No.	Chemical Structure
**EH-1**	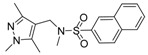	**5**	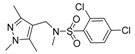
**1**	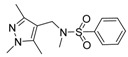	**6**	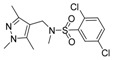
**2**	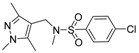	**7**	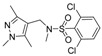
**3**	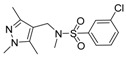	**8**	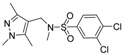
**4**	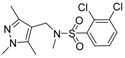	**9**	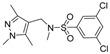

**Table 2:** RNA-Seq and qRT-PCR analysis of select genes up-regulated by **EH-1** and/or ACC.

**A. Genes Up-Regulated by EH-1**				
**Gene ID**	**Gene**	**Fold Change RNA-Seq (EH-1/Cont)**	**Fold Change qRT-PCR (EH-1/Cont)**	**Name of the Gene**
***AT1G72290***	***AT1G72290***	**134.32**	**97.13 ± 49.08**	**At1g72290; T9N14.19**
*AT1G15520*	*ABCG40*	69.63	33.51 ± 15.26	ABCG40
*AT4G12870*	*AT4G12870*	48.45	23.36 ± 15.19	GILT reductase family protein
*AT1G17180*	*GSTU25*	46.71	14.65 ± 7.47	Glutathione S-transferase U25
*AT3G22640*	*PAP85*	41.20	39.69 ± 17.62	AT3g22640/MWI23_1
*AT5G44120*	*CRA1*	36.33	47.93 ± 18.96	12S seed storage protein CRA1
**B. Genes Up-Regulated by ACC**				
**Gene ID**	**Gene**	**Fold Change RNA-Seq (ACC/Cont)**	**Fold Change qRT-PCR (ACC/Cont)**	**Name of the Gene**
*AT5G42530*	*AT5G42530*	103.72	78.96 ± 12.28	At5g42530
***AT1G72290***	***AT1G72290***	**85.83**	73.50 ± 24.30	**At1g72290**
*AT4G12480*	*EARLI1*	48.43	49.84 ± 13.56	At1g72290
*AT2G41230*	*ORS1*	47.02	35.98 ± 2.72	Lipid transfer protein EARLI 1
*AT2G05510*	*AT2G05510*	44.77	43.67 ± 11.78	Protein ORGAN SIZE RELATED 1
*AT5G21120*	*EIL2*	32.40	3.99 ± 2.64	At2g05510

Data obtained from RNA-seq were estimated by three line of independent samples using software as indicated in the experimental. qRT-PCR analysis were carried out as shown in the experiment section and all the experiments were done three times to establish the repeatability.

## Supplementary Materials

Supplementary materials are available online.
