# Supplementary material for: A Chemical Genetics Strategy That Identifies Small Molecules Which Induce the Triple Response in Arabidopsis
Source: Molecules. 2017 Dec 19;22(12):2270. doi: 10.3390/molecules22122270 (PMC6149847; doi:10.3390/molecules22122270)
Supplement: Supplementary file 1 [file molecules-22-02270-s001.pdf]

Supplementary Materials

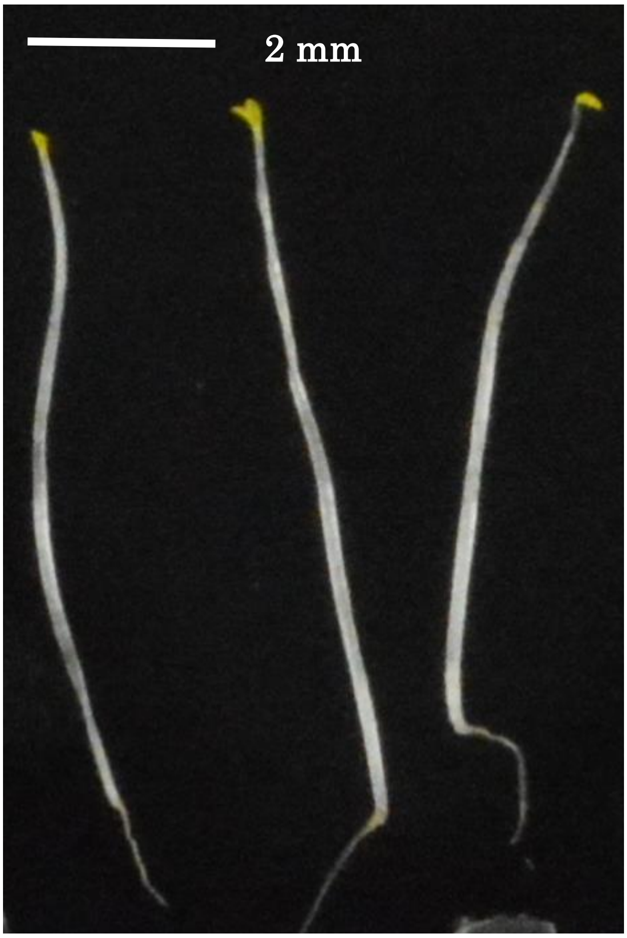

Figure S1. DMSO Mock-teated Arabidopsis seedlings

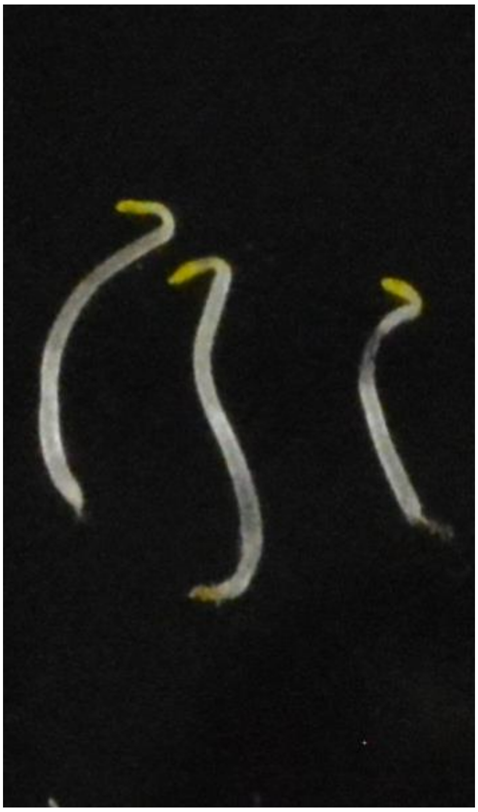

Figure S2. ACC (10 μM)

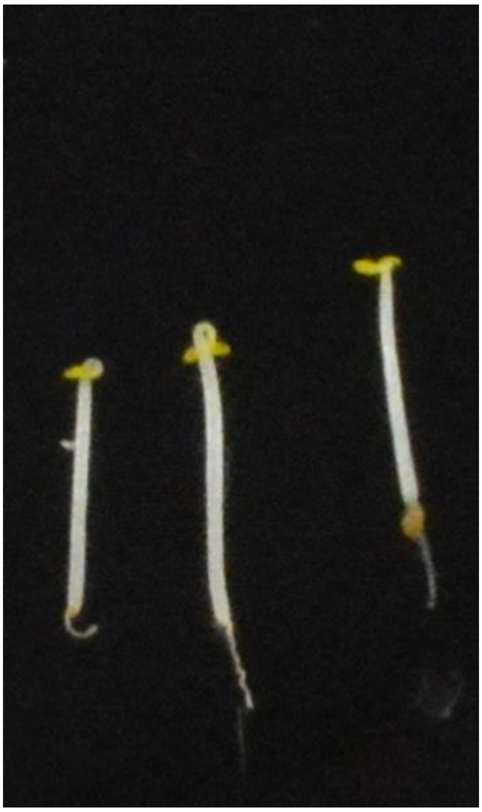

Figure S3. EH-1(10 μM)

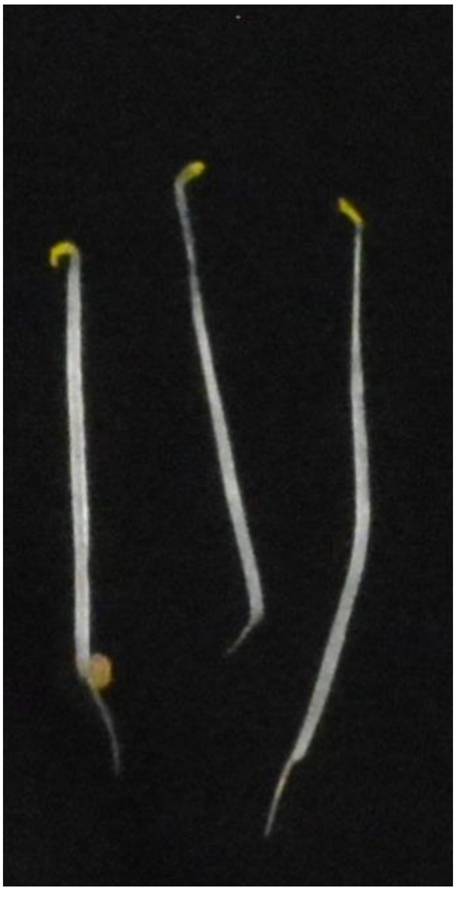

Figure S4. *N-Methyl-N-[(1, 3, 5-trimethyl-1H-pyrazol-4-yl) methyl] benzenesulfonamide* (***1***)*:*

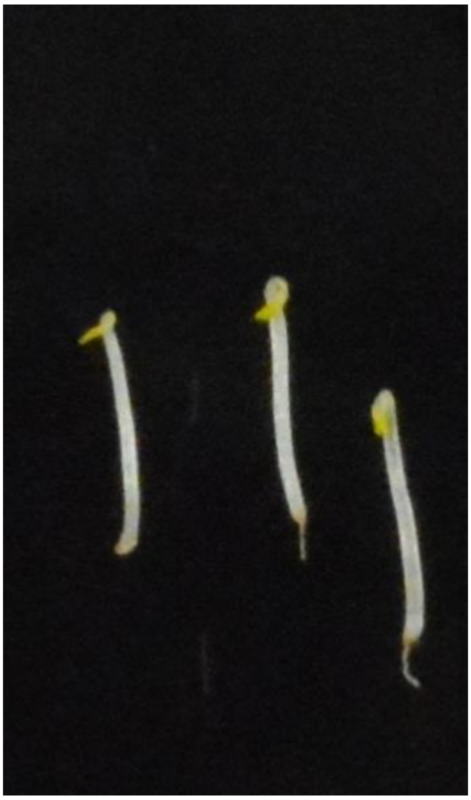

Figure S5. *4-Chloro-N-methyl-N-[(1, 3, 5-trimethyl-1H-pyrazol-4-yl) methyl benzenesulfonamide (***2***):*

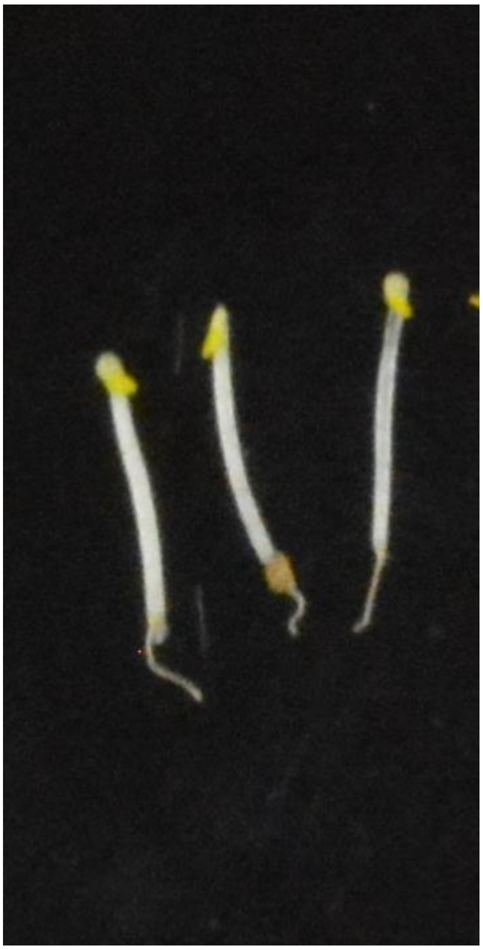

Figure S6. *3-Chloro-N-methyl-N-[(1, 3, 5-trimethyl-1H-pyrazol-4-yl) methyl benzenesulfonamide (***3***):*

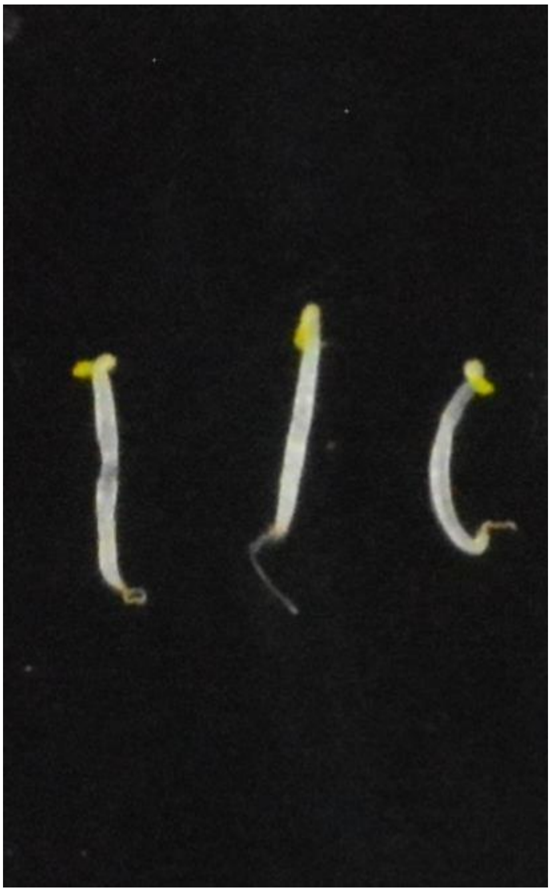

Figure S7. *2, 3-Dichloro-N-methyl-N-(1, 3, 5-trimethyl-1H-pyrazol-4-ylmethyl)-benzenesulfonamide (***4***):*

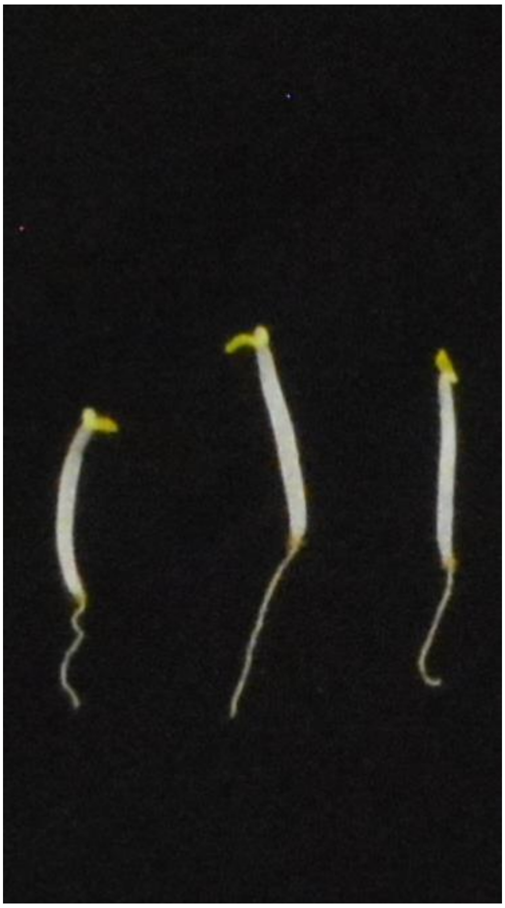

Figure S8. *2, 4-Dichloro-N-methyl-N-(1, 3, 5-trimethyl-1H-pyrazol-4-ylmethyl)benzenesulfonamide (***5***):*

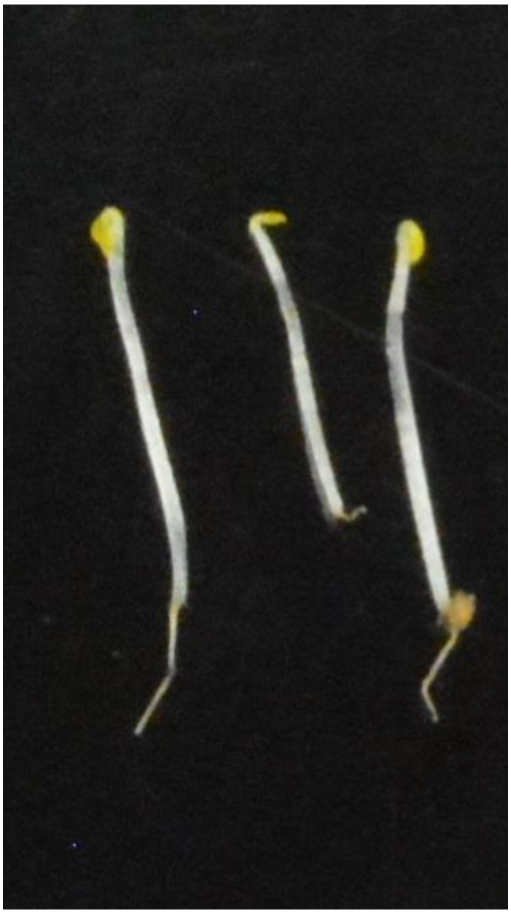

Figure S9. *2, 5-Dichloro-N-methyl-N-(1, 3, 5-trimethyl-1H-pyrazol-4-ylmethyl)-benzenesulfonamide (***6***):*

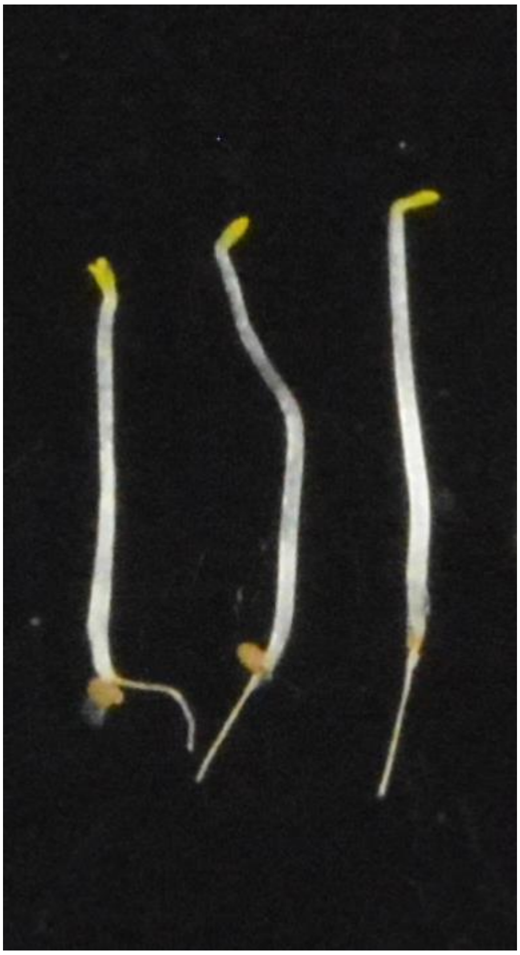

Figure S10. *2, 6-Dichloro-N-methyl-N-(1, 3, 5-trimethyl-1H-pyrazol-4-ylmethyl)benzenesulfonamide (***7***):*

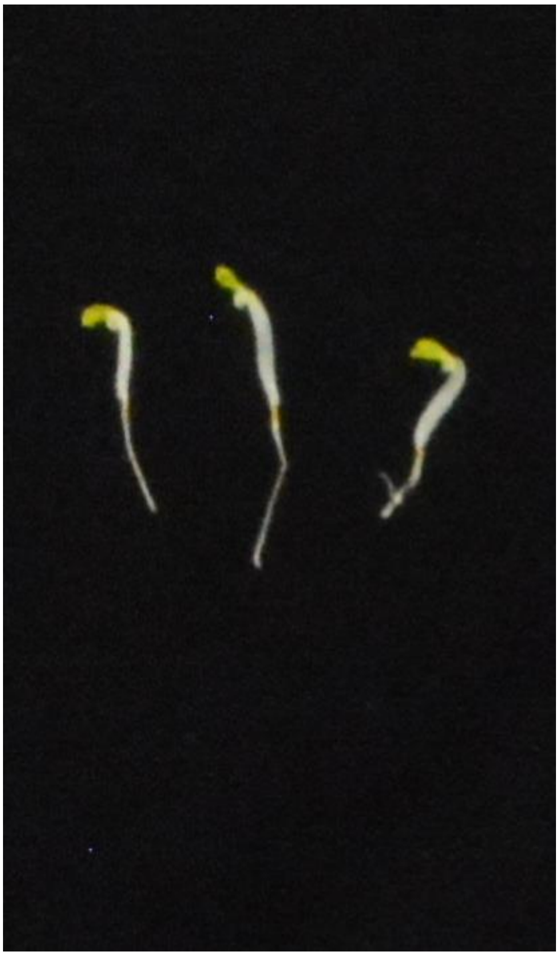

Figure S11. *3, 4-Dichloro-N-methyl-N-(1, 3, 5-trimethyl-1H-pyrazol-4-ylmethyl)benzenesulfonamide (**8**):*

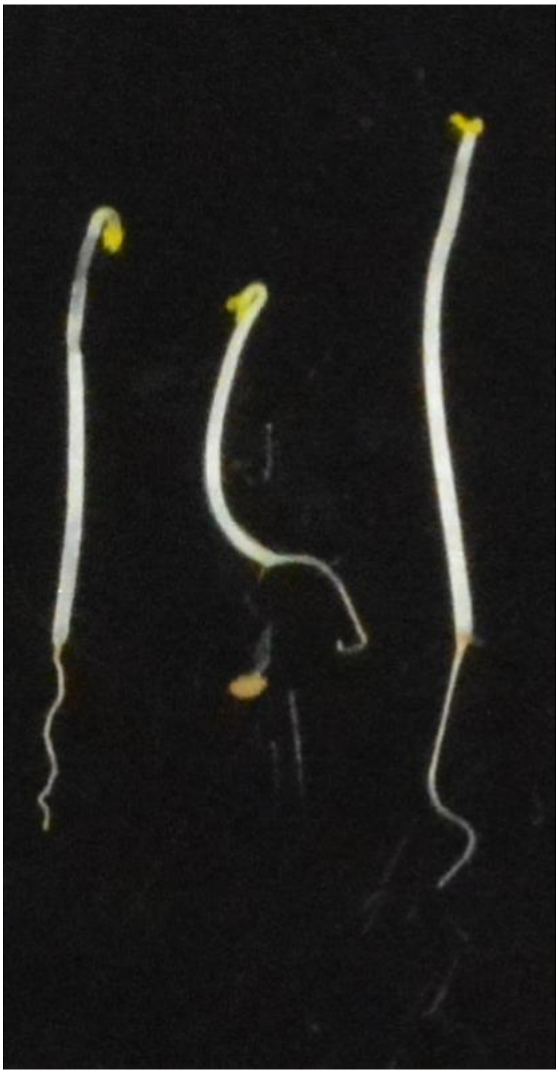

Figure S12. *3, 5-Dichloro-N-methyl-N-(1, 3, 5-trimethyl-1H-pyrazol-4-ylmethyl)benzenesulfonamide (**9**):*

Table S1. Annotation of A. thaliana genes differentially expressed by treatment of EH-1 or ACC.

Fold change (FC) is relative to non-treated condition (Cont), FC ≥20 for upregulation or ≤ 1/20 for downregulation, FDR-corrected P-value ≤ 0.05.

In the case of no gene expression (FPKM = 0) in one condition, highly expressed genes (FPKM ≥2) in the other condition are also qualified for upregulation or downretulation.

Common up- and down-regulated genes to both EH-1 and ACC treatment are shown as bold. The genes used for validation by qRT-PCR are shown as italic.

**Up-regulated genes by EH-1**					
**gene id**	**gene**	**FPKM (Cont)**	**FPKM (EH-1)**	**FC (EH-1/Cont)**	**Gene Name/Gene Symbol/Ortholog**
AT1G72290	***AT1G72290***	***0.253204***	***34.0101***	***134.3189681***	***At1g72290;T9N14.19;ortholog***
AT1G15520	*ABCG40*	*0.0556888*	*3.87749*	*69.62782463*	*ABC transporter G family member 40;ABCG40;ortholog*
AT4G01490	AT4G01490	0.052938	3.20703	60.58086819	-
AT2G47520	*ERF071*	*0.384704*	*20.7729*	*53.99709907*	*Ethylene-responsive transcription factor ERF071;ERF071;ortholog*
AT4G12870	*AT4G12870*	*0.239607*	*11.6094*	*48.45183989*	*Gamma interferon responsive lysosomal thiol (GILT) reductase family protein;At4g12870;ortholog*
AT1G17180	*GSTU25*	*1.31261*	*61.3155*	*46.71265646*	*Glutathione S-transferase U25;GSTU25;ortholog*
AT3G22640	*PAP85*	*2.73366*	*112.623*	*41.19861285*	*AT3g22640/MWI23_1;PAP85;ortholog*
AT4G16870	AT4G16870	0.159938	6.47814	40.50407033	-
**AT4G25860**	**ORP4A**	**0.0690631**	**2.68396**	**38.8624316**	**Oxysterol-binding protein-related protein 4A;ORP4A;ortholog**
AT5G44120	*CRA1*	*31.0836*	*1129.14*	*36.3259082*	*12S seed storage protein CRA1;CRA1;ortholog*
AT4G16860	RPP4	0.252082	8.37902	33.23926341	Disease resistance protein RPP4;RPP4;ortholog
AT2G26150	HSFA2	0.727522	22.771	31.29939713	Heat stress transcription factor A-2;HSFA2;ortholog
**AT4G34419**	**AT4G34419**	**0.592671**	**18.4238**	**31.08604943**	**-**
AT1G77950	AGL67	0.311286	9.43306	30.3035151	Protein agamous-like 67;AGL67;ortholog
AT1G51820	AT1G51820	0.0305168	0.924159	30.28361427	Probable LRR receptor-like serine/threonine-protein kinase At1g51820;At1g51820;ortholog
AT5G51440	HSP23.5	2.14007	64.0886	29.94696435	23.5 kDa heat shock protein, mitochondrial;HSP23.5;ortholog
AT1G65340	CYP96A3	0.151558	4.50014	29.69252695	Cytochrome P450, family 96, subfamily A, polypeptide 3;T8F5.12;ortholog
AT5G39180	AT5G39180	0.12293	3.25096	26.44561946	Germin-like protein subfamily 1 member 19;At5g39180;ortholog
AT1G14930	AT1G14930	0.921865	24.0328	26.06976076	Major latex homologue type2;MLP2;ortholog
AT2G24560	AT2G24560	0.0784361	1.99446	25.42783234	GDSL esterase/lipase At2g24560;At2g24560;ortholog
AT1G02065	SPL8	0.0532371	1.34666	25.2955176	Squamosa promoter-binding-like protein 8;SPL8;ortholog
**AT1G56680**	**AT1G56680**	**0.19715**	**4.80128**	**24.35343647**	**Chitinase family protein;F25P12.88;ortholog**
AT5G07200	GA20OX3	0.119717	2.90602	24.2740797	Gibberellin 20 oxidase 3;GA20OX3;ortholog
AT4G35200	AT4G35200	0.124226	2.97251	23.92824368	Putative uncharacterized protein AT4g35200;T12J5.70;ortholog
AT1G68250	AT1G68250	6.74766	154.396	22.8814137	At1g68250;T22E19.12;ortholog
AT2G23630	sks16	0.0538461	1.20553	22.38843667	Protein SKU5 similar 16;sks16;ortholog
AT5G13170	SWEET15	0.0676881	1.45062	21.43094576	Bidirectional sugar transporter SWEET15;SWEET15;ortholog
AT5G62340	AT5G62340	0.943527	20.0327	21.23171886	At5g62340;At5g62340;ortholog
AT1G76680	OPR1	19.7639	405.733	20.52899478	12-oxophytodienoate reductase 1;OPR1;ortholog
AT1G61275	U12	0	11.5062		-
AT1G08005	AT1G08005	0	6.1123		Uncharacterized protein;At1g08005;ortholog
**AT5G59040**	**COPT3**	**0**	**5.85884**		**Copper transporter 3;COPT3;ortholog**
AT2G36750	UGT73C1	0	4.09267		UDP-glycosyltransferase 73C1;UGT73C1;ortholog
AT2G35743	AT2G35743	0	3.96511		-
AT2G07825	AT2G07825	0	2.98114		Uncharacterized protein;At2g07825;ortholog
AT3G26200	CYP71B22	0	2.9791		Cytochrome P450 71B22;CYP71B22;ortholog
AT2G33790	AGP30	0	2.82685		Non-classical arabinogalactan protein 30;AGP30;ortholog
AT3G54530	AT3G54530	0	2.30046		Uncharacterized protein;At3g54530;ortholog
AT4G05091	AT4G05091	0	2.17651		Uncharacterized protein;At4g05091;ortholog

Table S1. *Cont.*

gene id	gene	FPKM (Cont)	FPKM (EH-1)	FC (EH-1/Cont)	Gene Name/Gene Symbol/Ortholog
**Down-regulated genes by EH-1**					
AT5G66260	AT5G66260	11.2997	0	0	Auxin-induced protein-like;At5g66260;ortholog
AT3G63088	RTFL14	10.3624	0	0	DVL14;DVL14;ortholog
**AT5G64770**	**RGF9**	**8.50822**	**0**	**0**	**Root meristem growth factor 9;RGF9;ortholog**
AT1G33640	AT1G33640	6.99401	0	0	Putative uncharacterized protein T1E4.1;T1E4.1;ortholog
AT4G10160	ATL59	4.13913	0	0	E3 ubiquitin-protein ligase ATL59;ATL59;ortholog
AT3G15440	AT3G15440	3.2653	0	0	Genomic DNA, chromosome 3, P1 clone: MJK13;MJK13.10;ortholog
AT5G43840	HSFA6A	3.1529	0	0	Heat stress transcription factor A-6a;HSFA6A;ortholog
AT3G02670	AT3G02670	3.06468	0	0	F16B3.30 protein;F16B3.30;ortholog
AT2G14160	AT2G14160	3.04342	0	0	RNA recognition motif-containing protein;At2g14160;ortholog
AT5G60480	AtHB26	2.28928	0	0	Zinc-finger homeodomain protein 12;ZHD12;ortholog
AT2G05440	ATGRP9	2.01758	0	0	Glycine-rich protein 9;T20G20.21;ortholog
AT4G37220	AT4G37220	419.705	0.329202	0.000784365	Cold-regulated 413 plasma membrane protein 4;At4g37220;ortholog
AT1G08630	THA1	229.657	0.342862	0.001492931	Probable low-specificity L-threonine aldolase 1;THA1;ortholog
**AT1G72140**	**NPF5.12**	**19.3252**	**0.0871194**	**0.004508072**	**Protein NRT1/ PTR FAMILY 5.12;NPF5.12;ortholog**
AT2G34430	LHB1B1	140.984	1.04861	0.007437794	Light-harvesting chlorophyll protein complex II subunit B1;Lhb1B1;ortholog
AT2G34490	CYP710A2	9.29248	0.126815	0.013647057	Cytochrome P450 710A2;CYP710A2;ortholog
AT2G32880	AT2G32880	11.5152	0.175397	0.015231781	TRAF-like family protein;At2g32880;ortholog
AT3G61060	PP2A13	235.275	3.90864	0.01661307	F-box protein PP2-A13;PP2A13;ortholog
AT3G55940	PLC7	9.06576	0.158371	0.017469137	Phosphoinositide phospholipase C 7;PLC7;ortholog
AT5G38710	POX2	7.17742	0.130346	0.018160565	Proline dehydrogenase 2, mitochondrial;POX2;ortholog
AT5G65800	ACS5	13.0255	0.240401	0.018456182	1-aminocyclopropane-1-carboxylate synthase 5;ACS5;ortholog
AT1G78230	AT1G78230	12.3287	0.235678	0.019116209	Outer arm dynein light chain 1 protein;At1g78230;ortholog
**AT4G11320**	**AT4G11320**	**628.663**	**12.5362**	**0.019941049**	**Probable cysteine proteinase At4g11320;At4g11320;ortholog**
**AT4G11190**	**DIR13**	**54.5116**	**1.12559**	**0.020648633**	**Dirigent protein 13;DIR13;ortholog**
AT5G47240	NUDT8	47.2855	0.990661	0.02095063	Nudix hydrolase 8;NUDT8;ortholog
AT1G15040	AT1G15040	184.394	3.91533	0.0212335	Putative glutamine amidotransferase;At1g15040;ortholog
**AT5G42600**	**MRN1**	**5.95423**	**0.131393**	**0.022067169**	**Marneral synthase;MRN1;ortholog**
AT1G33813	AT1G33813	3.0628	0.0735831	0.024024781	-
AT5G63450	CYP94B1	22.0556	0.542915	0.024615744	Cytochrome P450 94B1;CYP94B1;ortholog
AT4G10830	AT4G10830	1.60184	0.0416424	0.025996604	-
AT4G24180	TLP1	12.031	0.313452	0.026053695	-
AT4G08360	AT4G08360	10.6937	0.27914	0.02610322	KOW domain-containing protein;T28D5.50;ortholog
AT1G49570	PER10	103.275	2.82276	0.027332462	Peroxidase 10;PER10;ortholog
AT1G10070	BCAT2	479.555	13.4898	0.028129829	Branched-chain-amino-acid aminotransferase 2, chloroplastic;BCAT2;ortholog
AT1G65310	XTH17	153.777	4.46764	0.029052719	Probable xyloglucan endotransglucosylase/hydrolase protein 17;XTH17;ortholog
AT2G43050	PME16	468.103	14.1141	0.030151697	Probable pectinesterase/pectinesterase inhibitor 16;PME16;ortholog
AT5G53190	SWEET3	26.3421	0.835575	0.031720136	Bidirectional sugar transporter SWEET3;SWEET3;ortholog
AT5G26146	AT5G26146	19.106	0.621082	0.032507171	-
AT3G14770	SWEET2	45.4448	1.48607	0.032700551	Bidirectional sugar transporter SWEET2;SWEET2;ortholog
AT4G19380	FAO4A	12.7514	0.419766	0.032919209	Long-chain-alcohol oxidase FAO4A;FAO4A;ortholog
AT2G20080	AT2G20080	21.2858	0.714955	0.033588355	Putative uncharacterized protein At2g20080/T2G17.12;At2g20080;ortholog
AT4G18510	CLE2	14.0054	0.476833	0.034046368	CLAVATA3/ESR (CLE)-related protein 2;CLE2;ortholog
AT5G56870	BGAL4	808.758	28.234	0.034910319	Beta-galactosidase 4;BGAL4;ortholog
AT2G29620	AT2G29620	5.44911	0.193894	0.035582691	Putative uncharacterized protein At2g29620;At2g29620;ortholog
AT3G03240	AT3G03240	4.4684	0.159658	0.035730463	Alpha/beta-Hydrolases superfamily protein;At3g03240;ortholog
AT1G19530	AT1G19530	1293.7	48.6815	0.037629667	Putative uncharacterized protein At1g19530;At1g19530;ortholog
AT5G52250	RUP1	19.3539	0.729816	0.037708989	WD repeat-containing protein RUP1;RUP1;ortholog
AT5G26150	AT5G26150	57.8409	2.18996	0.03786179	Protein kinase;At5g26150;ortholog
AT1G69490	NAC029	44.624	1.69265	0.037931382	NAC transcription factor 29;NAC029;ortholog
AT4G28140	ERF054	2.24682	0.0864023	0.038455372	Ethylene-responsive transcription factor ERF054;ERF054;ortholog

Table S1. *Cont.*

AT4G25580	AT4G25580	171.105	6.92758	0.040487303	ARM repeat superfamily protein;At4g25580;ortholog
AT5G24580	AT5G24580	38.6006	1.58798	0.041138739	Heavy metal transport/detoxification domain-containing protein;At5g24580;ortholog
AT1G15380	AT1G15380	208.356	8.73675	0.041931838	AT1G15380 protein;F9L1.33;ortholog
AT4G13310	CYP71A20	15.8383	0.681076	0.043001837	Cytochrome P450 71A20;CYP71A20;ortholog
**AT1G04660**	**AT1G04660**	**9.48056**	**0.407925**	**0.043027522**	**Glycine-rich protein;T1G11.8;ortholog**
AT2G25160	CYP82F1	7.24054	0.317219	0.043811511	At2g25160;CYP82F1;ortholog
AT3G51760	AT3G51760	1.52545	0.0669107	0.043862926	Uncharacterized protein;At3g51760;ortholog
AT1G43590	AT1G43590	5.2592	0.236959	0.045056092	-
AT2G28780	AT2G28780	39.5553	1.79675	0.045423749	Putative uncharacterized protein At2g28780;At2g28780;ortholog
AT5G44130	FLA13	66.1992	3.09813	0.046800112	Fasciclin-like arabinogalactan protein 13;FLA13;ortholog
AT3G15450	AT3G15450	3731.72	180.127	0.048269163	AT3g15450/MJK13_11;MJK13.11;ortholog
AT1G29395	COR413IM1	63.1282	3.05771	0.048436515	Cold-regulated 413 inner membrane protein 1, chloroplastic;COR413IM1;ortholog
AT1G80160	AT1G80160	111.854	5.44856	0.048711356	AT1G80160 protein;At1g80160;ortholog
AT2G22860	PSK2	75.7852	3.69794	0.048795015	Phytosulfokines 2;PSK2;ortholog
AT5G65690	PCK2	5.22065	0.255371	0.048915556	At5g65690;PCK2;ortholog
**AT2G29130**	**LAC2**	**5.05605**	**0.251826**	**0.049806865**	**Laccase-2;LAC2;ortholog**
AT4G16000	AT4G16000	26.8945	1.34438	0.049987172	Putative uncharacterized protein At4g16000;At4g16000;ortholog

Table S1. *Cont.*

**Up-regulated genes by ACC**					
**gene id**	**gene**	**FPKM (Cont)**	**FPKM (ACC)**	**FC (ACC/Cont)**	**Gene Name/Gene Symbol/Ortholog**
AT5G25180	CYP71B14	0.134684	15.1543	112.5174482	Cytochrome P450 71B14;CYP71B14;ortholog
AT5G42530	*AT5G42530*	*1.69907*	*176.235*	*103.7243904*	*Putative uncharacterized protein At5g42530;MDH9.23;ortholog*
AT1G19900	AT1G19900	0.131098	12.9601	98.85810615	At1g19900/F6F9_4;At1g19900;ortholog
AT1G72290	***AT1G72290***	***0.253204***	***21.7322***	***85.82881787***	***At1g72290;T9N14.19;ortholog***
AT2G41231	AT2G41231	4.46564	251.096	56.22844654	Uncharacterized protein;At2g41231;ortholog
AT3G49960	PER35	0.15229	7.97829	52.38879769	Peroxidase 35;PER35;ortholog
AT4G12480	*EARLI1*	*1.81684*	*87.9847*	*48.42732437*	*Lipid transfer protein EARLI 1;EARLI1;ortholog*
AT5G22410	PER60	0.0669309	3.17134	47.38230025	Peroxidase 60;PER60;ortholog
AT2G41230	*ORS1*	*1.09653*	*51.5585*	*47.01968938*	*Protein ORGAN SIZE RELATED 1;ORS1;ortholog*
AT3G29410	TPS25	0.251281	11.5188	45.84031423	Terpenoid synthase 25;TPS25;ortholog
AT2G05510	*AT2G05510*	*4.44297*	*198.909*	*44.76937724*	*At2g05510;At2g05510;ortholog*
AT4G34380	AT4G34380	0.108317	4.80891	44.39663211	At4g34380;At4g34380;ortholog
AT3G45280	SYP72	0.570371	24.4669	42.89646563	Syntaxin-72;SYP72;ortholog
AT5G04960	PME46	0.0843975	3.54471	42.00017773	Probable pectinesterase/pectinesterase inhibitor 46;PME46;ortholog
AT5G14200	IMDH3	0.0764425	3.05773	40.00039245	3-isopropylmalate dehydrogenase 3, chloroplastic;IMDH3;ortholog
AT4G25220	RHS15	0.0577305	2.09335	36.26072873	Putative glycerol-3-phosphate transporter 2;At4g25220;ortholog
AT4G08410	AT4G08410	0.040638	1.43364	35.27831094	Extensin-like protein;T28D5.100;ortholog
AT5G01180	NPF8.2	0.210113	7.22647	34.39325506	Protein NRT1/ PTR FAMILY 8.2;NPF8.2;ortholog
AT3G49970	AT3G49970	0.291062	9.50551	32.65802475	Putative BTB/POZ domain-containing protein At3g49970;At3g49970;ortholog
AT5G21120	*EIL2*	*0.0591848*	*1.91744*	*32.39750747*	*ETHYLENE INSENSITIVE 3-like 2 protein;EIL2;ortholog*
AT2G24980	AT2G24980	0.22421	7.25879	32.37496097	Proline-rich extensin-like family protein;At2g24980;ortholog
**AT1G56680**	**AT1G56680**	**0.19715**	**6.14957**	**31.19234086**	**Chitinase family protein;F25P12.88;ortholog**
**AT4G25860**	**ORP4A**	**0.0690631**	**2.10259**	**30.44447759**	**Oxysterol-binding protein-related protein 4A;ORP4A;ortholog**
AT5G35190	AT5G35190	0.0909521	2.65967	29.24253536	Proline-rich extensin-like family protein;At5g35190;ortholog
AT1G12560	EXPA7	0.258061	7.35786	28.51209598	Expansin-A7;EXPA7;ortholog
AT4G20210	TPS08	0.0455544	1.26968	27.87173138	Terpenoid synthase 8;TPS08;ortholog
AT1G23510	AT1G23510	0.240402	6.51744	27.11058976	Uncharacterized protein;At1g23510;ortholog
AT4G13390	AT4G13390	1.59161	40.7449	25.59980146	Extensin-like protein;At4g13390;ortholog
AT4G09820	TT8	0.0477894	1.21632	25.4516692	Transcription factor TT8;TT8;ortholog
AT3G10710	PME24	0.0981318	2.43114	24.77423221	Putative pectinesterase/pectinesterase inhibitor 24;PME24;ortholog
AT3G27650	LBD25	0.448499	10.613	23.66337495	LOB domain-containing protein 25;LBD25;ortholog
AT1G11250	SYP125	0.255654	5.91219	23.12574808	Syntaxin-125;SYP125;ortholog
AT5G63660	PDF2.5	1.1678	26.7157	22.87694811	Defensin-like protein 6;PDF2.5;ortholog
AT4G13210	AT4G13210	0.372739	8.43824	22.6384682	Putative pectate lyase 14;At4g13210;ortholog
AT1G65570	AT1G65570	0.181173	4.07043	22.46708947	F5I14.10;F5I14.10;ortholog
**AT4G34419**	**AT4G34419**	**0.592671**	**13.1506**	**22.18870166**	**-**
AT5G50260	CEP1	0.300735	6.64547	22.09742797	KDEL-tailed cysteine endopeptidase CEP1;CEP1;ortholog
AT5G16080	CXE17	0.578221	12.3049	21.28061762	Probable carboxylesterase 17;CXE17;ortholog
AT4G25820	XTH14	0.918858	18.6643	20.3124966	Xyloglucan endotransglucosylase/hydrolase protein 14;XTH14;ortholog
AT4G11393	AT4G11393	0	14.2396		Putative defensin-like protein 202;At4g11393;ortholog
AT3G28320	AT3G28320	0	6.53089		UPF0496 protein At3g28310/At3g28320;At3g28310/At3g28320;ortholog
AT4G28850	XTH26	0	3.4647		Probable xyloglucan endotransglucosylase/hydrolase protein 26;XTH26;ortholog
AT3G45180	AT3G45180	0	3.09638		Putative uncharacterized protein T14D3.120;T14D3.120;ortholog
AT5G48205	AT5G48205	0	2.72018		Zinc ion binding protein;At5g48205;ortholog
**AT5G59040**	**COPT3**	**0**	**2.37833**		**Copper transporter 3;COPT3;ortholog**
AT5G09370	AT5G09370	0	2.23659		Bifunctional inhibitor/lipid-transfer protein/seed storage 2S albumin superfamily protein;T5E8_170;ortholog
AT1G63040	AT1G63040	0	2.21646		Ethylene-responsive transcription factor ERF026;ERF026;ortholog
AT2G20160	ASK17	0	2.14043		SKP1-like protein 17;ASK17;ortholog

Table S1. *Cont.*

Down-regulated genes by ACC					
**gene id**	**gene**	**FPKM (Cont)**	**FPKM (ACC)**	**FC (ACC/Cont)**	**Gene Name/Gene Symbol/Ortholog**
ATMG01380	RRN5	254.13	0	0	-
AT1G31258	AT1G31258	48.7533	0	0	-
AT2G16005	AT2G16005	14.4116	0	0	At2g16001;At2g16005;ortholog
**AT5G64770**	**RGF9**	**8.50822**	**0**	**0**	**Root meristem growth factor 9;RGF9;ortholog**
AT3G53232	RTFL1	5.38111	0	0	At3g53232;DVL20;ortholog
AT4G11210	DIR14	3.17402	0	0	Dirigent protein 14;DIR14;ortholog
**AT1G72140**	**NPF5.12**	**19.3252**	**0.112495**	**0.005821156**	**Protein NRT1/ PTR FAMILY 5.12;NPF5.12;ortholog**
AT4G12550	AIR1	87.7245	0.654674	0.007462841	Putative lipid-binding protein AIR1;AIR1;ortholog
AT1G73330	ATDR4	41.3669	0.310701	0.00751086	Dr4 protein;Dr4;ortholog
**AT1G04660**	**AT1G04660**	**9.48056**	**0.0734922**	**0.007751884**	**Glycine-rich protein;T1G11.8;ortholog**
AT5G13930	CHS	10.8893	0.1582	0.014528023	Chalcone synthase;CHS;ortholog
AT5G47990	CYP705A5	20.7681	0.324707	0.015634892	Cytochrome P450 705A5;CYP705A5;ortholog
AT5G37300	WSD1	5.81421	0.0998712	0.017177089	O-acyltransferase WSD1;WSD1;ortholog
**AT4G11190**	**DIR13**	**54.5116**	**0.988492**	**0.018133608**	**Dirigent protein 13;DIR13;ortholog**
**AT2G29130**	**LAC2**	**5.05605**	**0.100771**	**0.019930776**	**Laccase-2;LAC2;ortholog**
AT5G15970	KIN2	36.987	0.759065	0.020522481	Stress-induced protein KIN2;KIN2;ortholog
AT4G14690	ELIP2	14.7367	0.362282	0.024583658	Early light-induced protein 2, chloroplastic;ELIP2;ortholog
AT5G53870	ENODL1	18.8684	0.47638	0.025247504	At5g53870;ENODL1;ortholog
AT4G13290	CYP71A19	4.58265	0.122959	0.026831419	Cytochrome P450 71A19;CYP71A19;ortholog
AT4G39480	CYP96A9	1.61001	0.0469053	0.029133546	Cytochrome P450, family 96, subfamily A, polypeptide 9;CYP96A9;ortholog
**AT4G11320**	**AT4G11320**	**628.663**	**21.4714**	**0.03415407**	**Probable cysteine proteinase At4g11320;At4g11320;ortholog**
AT5G48010	THAS1	2.8592	0.104122	0.03641648	Thalianol synthase;THAS1;ortholog
AT5G57785	AT5G57785	49.3792	1.9612	0.039717128	At5g57785;At5g57785;ortholog
**AT5G42600**	**MRN1**	**5.95423**	**0.254451**	**0.042734493**	**Marneral synthase;MRN1;ortholog**
AT3G51240	F3H	2.09869	0.0902045	0.042981336	Naringenin,2-oxoglutarate 3-dioxygenase;F3H;ortholog
AT1G66800	AT1G66800	6.53514	0.291072	0.04453952	Alcohol dehydrogenase-like protein;At1g66800;ortholog
AT2G39250	SNZ	5.17442	0.233676	0.045159844	AP2-like ethylene-responsive transcription factor SNZ;SNZ;ortholog
AT3G06880	AT3G06880	1.58653	0.0728162	0.045896516	Transducin/WD-40 repeat-containing protein;At3g06880;ortholog
AT4G29030	AT4G29030	3.33883	0.15336	0.045932258	Glycine-rich protein like;F19B15.60;ortholog
AT1G56600	GOLS2	1.48668	0.0695491	0.046781486	Galactinol synthase 2;GOLS2;ortholog
AT1G29910	LHCB1.2	45.6284	2.19161	0.048031708	Chlorophyll a-b binding protein 3, chloroplastic;LHCB1.2;ortholog
AT5G16030	AT5G16030	48.5212	2.33452	0.048113402	Uncharacterized protein;At5g16030;ortholog

Table S2. PCR primers used in this study.

Upregulated by EH-1 treatment

AGI	Gene Name	Forwerd primers 5'-3'	Reverse primers 5'-3'	Reference
At1g72290	Kunitz-PI;1	ACCGGTGAAGGATACAGCCG	TGTAATGGCGGCTGGGACAA	Boex-Fontvieille et al., 2016
At1g15520	PDR12	GAGTGGACGGCGGTGAAG	TGGGACGAGGGATGAGGAA	Jiang et al., 2017
At2g47520	AtERF71/HRE2	AGTAGAGAGCTTATCGGAGGACCTG	TTCCAATATCGGTCGCCGATTGGTC	Lee et al., 2015
At4g12870	GILT	GGTGGGTCAAGTCTGCAAAGC	TTCCTCTCGGACAAGGCAGC	Ohkama-Ohtsu et al., 2004
At1g17180	GST-U25	TGTCAAATTCGATTACAGAGAACAAG	GGTATTTTCTTATGAACCGGATTCA	Gunning et al., 2014
At5g44120	CRA1	AGCCCAAATCCAGATCGTAAAC	TCACCACCGAGAAACCTTGTG	Gao et al., 2016
At3g22640	PAP85	CTTCACATAGCCGCCGCTTG	CATCTCGAAGCGGGCACAAC	Chen et al., 2013

Upregulated by ACC treatment

AGI	Gene Name	Forwerd primers 5'-3'	Reverse primers 5'-3'	Reference
At5g42530	SCSG1	TCAATGACCAGGTTCGAGTG	CAAGAGGAAGCCCAGAAACA	Raissig et al., 2013
At4g12480	EARLI1	TTTGTCTTTGCACTGCGCTTAGGG	TTGGAAGCCAGACGGAACCTTTCT	Xu et al., 2011
At2g41230	OSR1	CTCTTTCTCTCTCTGCTTCTTCTGATT	CCGGAGGCGGTGGAA	Feng et al., 2011
At2g05510	GRP1	TGATTCTGTTGGGTCTCTTCG	TTGCACAGTTGCCTCACT	Yang et al., 2017
At5g21120	EIL2	TGATAGGAACGGCCCAGCTG	TCCCCAACCTCAGACCCTGA	Camehl et al., 2010

Supplemental References

Boex-Fontvieille E.; Rustgi, S.; von Wettstein, D.; Pollmann, S.; Reinbothe, S.; Reinbothe C. An Ethylene-Protected Achilles' Heel of Etiolated Seedlings for Arthropod Deterrence. *Front. Plant Sci.* **2016**, 7, 1246.

Camehl, I.; Sherameti, I.; Venus, Y.; Bethke, G.; Varma, A.; Lee, J.; Oelmüller, R. Ethylene signalling and ethylene-targeted transcription factors are required to balance beneficial and nonbeneficial traits in the symbiosis between the endophytic fungus *Piriformospora indica* and *Arabidopsis thaliana*. *New Phytol.* **2010**, 185, 1062-73.

Chen, C. E.; Yeh, K. C.; Wu, S. H.; Wang, H. I.; Yeh, H. H. A vicilin-like seed storage protein, PAP85, is involved in tobacco mosaic virus replication. *J Virol.* **2013**, 87, 6888-900.

Feng, G.; Qin, Z.; Yan, J.; Zhang, X.; Hu, Y. *Arabidopsis* ORGAN SIZE RELATED1 regulates organ growth and final organ size in orchestration with ARGOS and ARL. *New Phytol.* **2011,** 191, 635-46.

Gao, C.; Qi, S.; Liu, K.; Li, D.; Jin, C.; Li, Z.; Huang, G.; Hai, J.; Zhang, M.; Chen, M. MYC2, MYC3, and MYC4 function redundantly in seed storage protein accumulation in *Arabidopsis*. *Plant Physiol. Biochem.* **2016**, 108, 63-70.

Gunning, V.; Tzafestas, K.; Sparrow, H.; Johnston, E. J.; Brentnall, A. S.; Potts, J. R.; Rylott, E. L.; Bruce, N.C. Arabidopsis Glutathione Transferases U24 and U25 Exhibit a Range of Detoxification Activities with the Environmental Pollutant and Explosive, 2,4,6-Trinitrotoluene. *Plant Physiol.* **2014**, 165, 854-865.

Jiang, L.; Wang, W.; Chen, Z.; Gao, Q.; Xu, Q.; Cao H. A role for *APX1* gene in lead tolerance in *Arabidopsis thaliana*. *Plant Sci.* **2017**, 256, 94-102.

Lee, S. Y.; Hwang, E. Y.; Seok, H. Y.; Tarte, V. N.; Jeong, M. S.; Jang, S. B.; Moon, Y. H. *Arabidopsis* AtERF71/HRE2 functions as transcriptional activator via *cis*-acting GCC box or DRE/CRT element and is involved in root development through regulation of root cell expansion. *Plant Cell Rep.* **2015**, 34, 223-231.

Ohkama-Ohtsu, N.; Kasajima, I.; Fujiwara, T.; Naito, S. Isolation and Characterization of an Arabidopsis Mutant That Overaccumulates O-Acetyl-L-Ser. *Plant Physiol.* **2004**, 136, 3209-3222.

Raissig, M. T.; Bemer, M.; Baroux, C.; Grossniklaus, U. Genomic Imprinting in the *Arabidopsis* Embryo Is Partly Regulated by PRC2. *PLoS Genet.* **2013**, 9, e1003862.

Xu, D.; Huang, X.; Xu, Z. Q.; Schläppi, M. The HyPRP gene *EARLI1* has an auxiliary role for germinability and early seedling development under low temperature and salt stress conditions in *Arabidopsis thaliana*. *Planta* **2011**, 234, 565-577.

Yang, Y.; La, H.; Tang, K.; Miki, D.; Yang, L.; Wang, B.; Duan, C. G.; Nie, W.; Wang, X.; Wang, S.; Pan, Y.; Tran, E. J.; An, L.; Zhang, H.; Zhu, J. K. SAC3B, a central component of the mRNA export complex TREX-2, is required for prevention of epigenetic gene silencing in *Arabidopsis*. *Nucleic Acids Res.*, **2017**, 45, 181–197.
